# Association of epicardial fat with left ventricular diastolic function in subjects with metabolic syndrome: assessment using 2-dimensional echocardiography

**DOI:** 10.1186/1471-2261-14-3

**Published:** 2014-01-09

**Authors:** Hyo Eun Park, Su-Yeon Choi, Minkyung Kim

**Affiliations:** 1 Division of Cardiology, Department of Internal Medicine, Healthcare System Gangnam Center, Seoul National University Hospital, 39th FL. Gangnam Finance Center, 737 Yeoksam-dong Gangnam-gu 135-984, Seoul, Korea

**Keywords:** Epicardial fat, Diastolic function, Metabolic syndrome

## Abstract

**Background:**

Metabolic syndrome (MetS) is related with left ventricular diastolic dysfunction (LVDD) and poor cardiovascular outcome. Epicardial adipose tissue (EAT) thickness, measured by echocardiography, is increased in subjects with MetS. However, the association of EAT with LV diastolic function has not been evaluated in subjects with MetS.

**Methods:**

In this retrospective study, EAT thickness was measured in 1,486 consecutive asymptomatic patients with no known heart disease who had transthoracic echocardiography during a self-referred healthcare exam. Subjects with a history of ischemic heart disease, cardiomyopathy or significant valvular heart disease were excluded. LVDD was defined as E/e’ ratio ≥ 15. Subjects were grouped into two groups, those with MetS and those without.

**Results:**

MetS was present in 346 subjects. There was no difference in LV systolic function between the two groups. However compared to patients without MetS, patients with MetS had larger left atrium (LA) size and higher E/e’ ratio (38 ± 5 versus 35 ± 5 mm for LA and 10.0 ± 3.3 versus 8.7 ± 2.7 for E/e’ ratio in subjects with versus without MetS both *p* < 0.001). LVDD was found in 27 (7.8%) subjects with MetS, compared to 30 (2.6%) subjects without MetS (*p* < 0.001). In subjects with MetS, EAT was significantly correlated with LVDD, even after adjusting for other cardiometabolic risk factors such as age, systolic blood pressure, BMI, blood glucose and LDL cholesterol (OR 1.845, 95% CI 1.153-2.951, *p* = 0.011).

**Conclusion:**

Greater EAT is found in subjects with MetS. EAT is significantly associated with LVDD in subjects with MetS, even after adjusting for other risk factors.

## Background

Epicardial adipose tissue (EAT) is a type of visceral adipose tissue (VAT) that surrounds the myocardium and epicardium. EAT has received much attention recently due to its metabolically active nature and relation with several bioactive adipokines [1-4]. Studies have shown an association between EAT and traditional cardiovascular risk factors, coronary atherosclerosis, coronary artery plaque burden, and major adverse cardiovascular events [5-7]. In addition to coronary artery disease, EAT is also correlated with left ventricular (LV) function [8,9]. However, the number of published studies and patients are both limited.

Increased EAT has been reported in patients with metabolic syndrome (MetS) [10,11]. MetS is a cluster of the well-established cardiovascular risk factors, including insulin resistance, obesity and related dyslipidemia. MetS is associated with poor cardiovascular outcome due to the atherogenic potential. The presence of MetS has also been linked with impaired LV diastolic function [12,13].

The association of EAT with either LV diastolic function or MetS have been separately studied. Yet, the association of EAT and LV diastolic function in the presence of MetS has not been studied in a large, asymptomatic population free of known coronary artery disease. Thus, we aimed to evaluate the correlation between EAT thickness measured by two dimensional echocardiography and LV diastolic function in a population of asymptomatic Koreans with MetS.

## Methods

### Study population

In this retrospective study, the study subjects were recruited from Seoul National University Hospital Healthcare System Gangnam Center. Among those who visited our echocardiography lab for a health screening exam between March 2011 and February 2012, EAT was measured by transthoracic echocardiography in 1,551 subjects without clinical signs and symptoms of heart disease. Those with significant valvular heart disease, LV systolic dysfunction, or cardiomyopathy were excluded from the analysis. Those without blood tests or whose clinical data were unavailable were also excluded from the analysis. In total, 1,486 asymptomatic subjects with EAT measured by echocardiography were included in this study. Past medical history and current medications were derived from self-reported medical questionnaires.

### Measurement of anthropometric parameters

All measurements were performed on the same day as the echocardiography. Height, body weight, waist circumference and blood pressure were measured. Waist circumference was measured in centimeters using the minimum circumference between the lower rib margin and the iliac crest in the standing position. Blood pressure was measured for three times in all patients with at least a 5 minutes interval between measures. Obesity was defined as a body mass index (BMI) ≥25 kg/m^2^[14], according to the modified WHO criteria from the Asia-Pacific guideline. Fasting blood glucose level, glycated hemoglobin, total cholesterol, triglyceride (TG), high density-lipoprotein (HDL) cholesterol, and low-density lipoprotein (LDL) cholesterol were measured after at least 12 hours of fasting in all patients.

MetS was classified according to the recommendations of National Cholesterol Education Program Adult Treatment Panel III [15]. Blood pressure ≥ 130/85 mmHg or the subjects’ self-reported history of hypertension or antihypertensive medications, a waist circumference ≥ 90 cm in men or ≥85 cm in women, triglyceride ≥ 150 mg/dL, HDL cholesterol ≤ 40 mg/dL in men and ≤50 mg/dL in women, fasting blood glucose level ≥ 100 mg/dL or a self-reported history of diabetes or use of diabetes medications were the parameters included to define MetS. When any three of the criteria met, the patient was defined as having MetS.

### Echocardiographic study

All study subjects underwent transthoracic echocardiography. Using commercially available equipment (Vivid 7, GE Medical Systems, Milwaukee, WI), an echocardiogram was performed by standard technique with subjects in the left lateral decubitus position. From the two-dimensional parasternal long axis view, epicardial fat was identified as the echo-free space between the outer wall of the myocardium and the visceral layer of the pericardium, and was measured perpendicularly on the right ventricular free wall at end-diastole [16].

Routine standard echocardiography exam parameters included LV end-diastolic and end-systolic dimensions, LV end-diastolic wall thickness, LV ejection fraction, pulsed-wave Doppler examination of the mitral inflow, and pulsed-wave tissue Doppler imaging at the medial mitral annulus. From the mitral inflow Doppler signals, early transmitral inflow velocity (E), late transmitral inflow velocity (A), and deceleration time (DT) of E velocity were obtained with the sample volume placed between the tips of mitral leaflets. Pulsed-wave tissue Doppler imaging-derived annular peak velocities (e’ and s’) were measured, and the ratio of mitral E peak velocity and e’ peak velocity (E/e’) was calculated. For assessment of LV diastolic function, E/e’ ratio was used, and subjects with E/e’ ≥ 15 were defined as having LV diastolic dysfunction (LVDD).

### Statistical analysis

Data are expressed as the mean ± standard deviation or frequency. Categorical variables were compared using chi-square analysis and continuous variables were compared using an unpaired Student’s t-test. To evaluate the correlation of the continuous parameters with LV diastolic function, bivariate correlation analysis with Pearson’s correlations was performed. To evaluate the correlation of various parameters with the presence of LVDD, regression analysis was performed. Using multiple regression analysis, other possible confounding covariables were adjusted. For all statistical analyses, statistical software package (IBM SPSS statistics 19.0) was used and a *p* < 0.05 was considered significant.

### Study ethics

The study protocol was reviewed and approved by the Institutional Review Board of Seoul National University Hospital (IRB No 1008-036-326). Because the current study was performed as a retrospective study using the database and medical records, informed consent was waived by the board.

## Results

The baseline characteristics are shown in Table 1. The mean age of the study population was 53 ± 9 years old, and 995 (67.0%) subjects were male. The mean systolic and diastolic blood pressures were 117 ± 13 mmHg and 77 ± 10 mmHg, respectively. The mean BMI was 24 ± 3 kg/m^2^ in the overall population. According to the criteria as described above, 346 subjects were defined as having MetS, and 1,140 subjects did not have MetS. The mean EAT was 2.7 ± 0.8 mm in the overall population. The mean LV ejection fraction was 66 ± 6% and the mean LA size was 40 ± 5 mm. LVDD was present in 57 (3.8%) subjects.

**Table 1:** Baseline characteristics

**Parameter**	**Total n = 1456**	**Subjects with metabolic syndrome n = 346**	**Subjects without metabolic syndrome n = 1140**	**p-value**
Male, n (%)	995 (67.0%)	267 (77.2%)	728 (63.9%)	<0.001
Age, yrs	53 ± 9	55 ± 10	53 ± 9	<0.001
SBP, mmHg	117 ± 13	125 ± 13	115 ± 12	<0.001
DBP, mmHg	77 ± 10	82 ± 10	75 ± 9	<0.001
PP, mmHg	40 ± 9	43 ± 10	40 ± 9	<0.001
BMI, kg/m^2^	24 ± 3	26 ± 3	23 ± 3	<0.001
WC, cm	87 ± 8	93 ± 6	85 ± 7	<0.001
Glucose, mg/dL	101 ± 21	116 ± 32	97 ± 14	<0.001
T. cholesterol, mg/dL	196 ± 33	198 ± 38	195 ± 32	0.157
Triglyceride, mg/dL	120 ± 81	188 ± 115	100 ± 52	<0.001
HDL cholesterol, mg/dL	51 ± 11	45 ± 9	53 ± 11	<0.001
LDL cholesterol, mg/dL	124 ± 29	126 ± 31	123 ± 29	0.101
hsCRP, mg/dL	0.15 ± 0.39	0.17 ± 0.32	0.14 ± 0.41	0.200
HbA1C, %	5.7 ± 0.7	6.1 ± 1.0	5.6 ± 0.5	<0.001
**Echocardiography**				
EAT, mm	2.7 ± 0.8	2.9 ± 0.9	2.7 ± 0.8	<0.001
LVIDd, mm	49 ± 4	50 ± 3	48 ± 4	<0.001
LVIDs, mm	28 ± 3	29 ± 3	28 ± 3	<0.001
LVEF,%	66 ± 6	66 ± 6	66 ± 6	0.526
LA, mm	40 ± 5	38 ± 5	35 ± 5	<0.001
E	0.59 ± 0.18	0.58 ± 0.27	0.60 ± 0.14	0.104
A	0.60 ± 0.25	0.65 ± 0.43	0.59 ± 0.16	0.004
DT	191 ± 60	198 ± 65	189 ± 58	0.014
E/A	1.0 ± 0.4	0.9 ± 0.3	1.1 ± 0.4	<0.001
E/E’	9.0 ± 2.9	10.0 ± 3.3	8.7 ± 2.7	<0.001
E/E’ ≥ 15	57 (3.8%)	27 (7.8%)	30 (2.6%)	<0.001

BMI; body mass index, DT; deceleration time, DBP; diastolic blood pressure, EAT; epicardial adipose tissue, HbA1C; glycated hemoglobin, HDL; high-density lipoprotein, hsCRP; high-sensitivity C-reactive protein, LA; left atrium size, LVIDd; left ventricular diastolic dimension, LVEF; left ventricular ejection fraction, LVIDs; left ventricular systolic dimension, LDL; low-density lipoprotein, PP; pulse pressure, SBP; systolic blood pressure, T.chol; total cholesterol, WC; waist circumference.

### Clinical and imaging parameters in subjects with and without MetS

The comparison analyses of the clinical and echocardiographic parameters are shown in Table 1. Among subjects with MetS, 267 (77.2%) were male, compared to 728 (63.9%) males in the population without MetS. The mean age, waist circumference and BMI were significantly higher in subjects with MetS (all *p* < 0.001). The systolic and diastolic blood pressure, fasting glucose level, glycated hemoglobin level and TG were significantly higher in the subjects with MetS (all *p* < 0.001).

**Figure 1:**
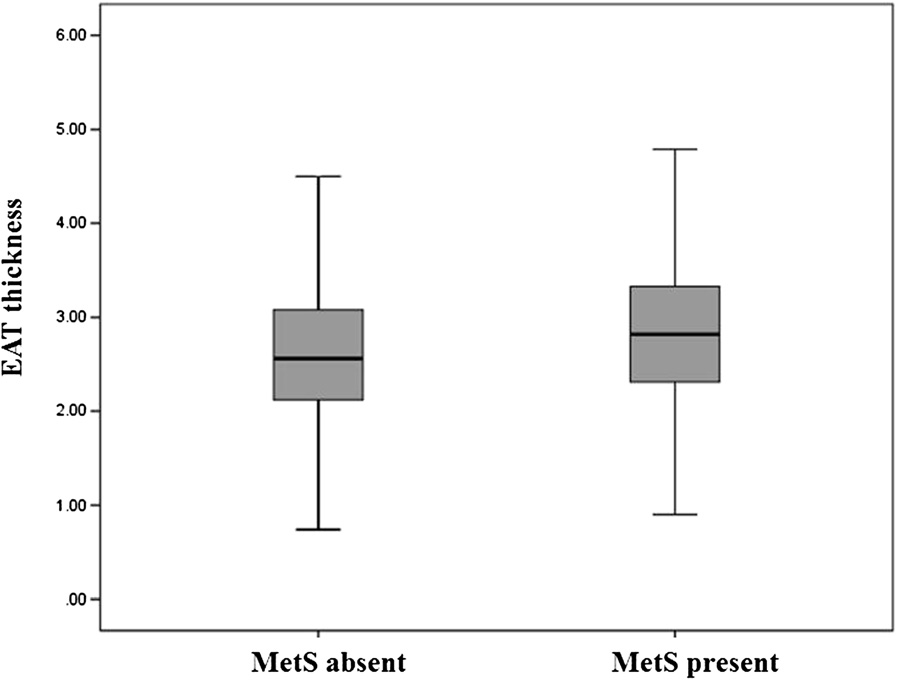
Epicardial fat thickness in subjects with versus without metabolic syndrome (Abbreviations: EAT = epicardial adipose tissue, MetS = metabolic syndrome).

**Figure 2:**
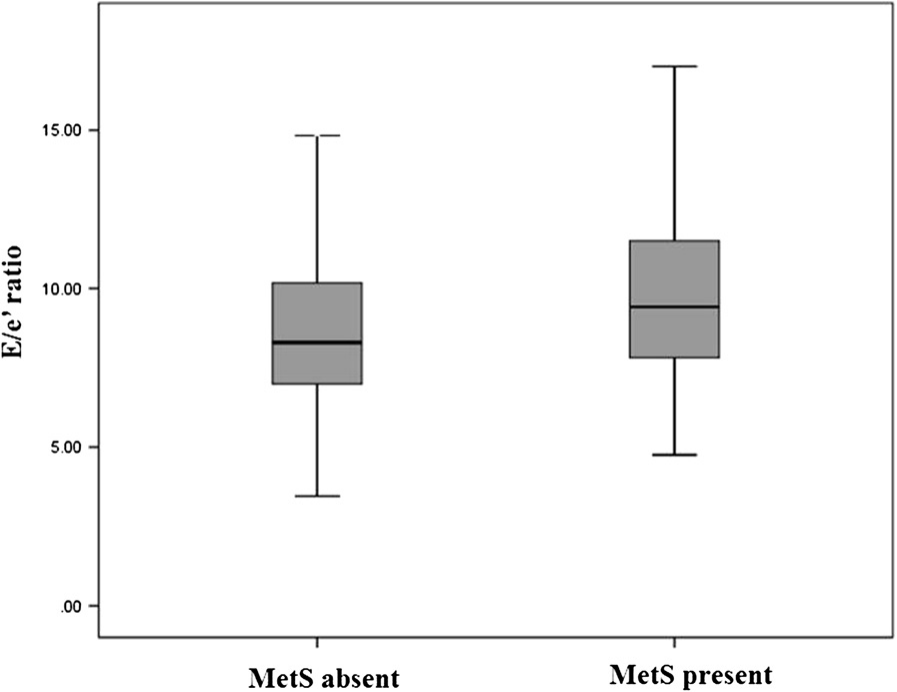
Left ventricular diastolic function in subjects with versus without metabolic syndrome (Abbreviations: MetS = metabolic syndrome).

The echocardiographic parameters showed no difference in LV systolic function between the two groups (*p* = 0.526). The mean EAT was greater in subjects with MetS compared to those without MetS (2.9 ± 0.9 versus (vs) 2.7 ± 0.8 mm in subjects with vs. without MetS, *p* < 0.001, Figure 1). Both a larger LA size and a higher E/e’ ratio were found in subjects with MetS versus those without MetS (38 ± 5 vs. 35 ± 5 mm and 10.0 ± 3.3 vs. 8.7 ± 2.7, respectively, both *p* < 0.001, Figure 2). LVDD was found in 27 (7.8%) subjects with MetS, compared to 30 (2.6%) subjects without MetS (*p* < 0.001).

### Correlation of clinical and imaging parameters with LV diastolic function

Bivariate analysis was performed to evaluate the association of various parameters with LV diastolic function (Table 2). In subjects with MetS, LV diastolic function as assessed by E/e’ ratio was significantly associated with age (*r* = 0.412, *p* < 0.001), systolic blood pressure (*r* = 0.296, *p* < 0.001), glycated hemoglobin level (*r* = 0.155, *p* = 0.004), LDL cholesterol (*r* = -0.112, *p* = 0.038), LA size (*r* = 0.266, *p* < 0.001) and EAT (*r* = 0.142, *p* = 0.008).

**Table 2:** Bivariate correlation analysis with E/E’ ratio

**Parameter**	**Patients with metabolic syndrome**		**Patients without metabolic syndrome**	
	**Correlation coefficient**	**p-value**	**Correlation coefficient**	**p-value**
Age	0.412	<0.001	0.447	<0.001
SBP	0.296	<0.001	0.194	<0.001
DBP	0.032	0.548	0.078	0.008
PP	0.345	<0.001	0.194	<0.001
BMI	0.027	0.620	0.188	<0.001
WC	-0.034	0.527	0.181	<0.001
Glucose	0.089	0.098	0.165	<0.001
HbA1C	0.155	0.004	0.146	<0.001
T. cholesterol	-0.087	0.107	-0.014	0.629
Triglyceride	-0.017	0.757	0.026	0.381
HDL cholesterol	0.032	0.547	0.019	0.514
LDL cholesterol	-0.112	0.038	-0.036	0.228
hsCRP	-0.070	0.277	0.072	0.053
EAT by TTE	0.142	0.008	0.041	0.166
LVEF	0.080	0.137	0.046	0.120
LA size	0.266	<0.001	0.273	<0.001

BMI; body mass index, DBP; diastolic blood pressure, EAT; epicardial adipose tissue, HbA1C; glycated hemoglobin, HDL; high-density lipoprotein, hsCRP; high-sensitivity C-reactive protein, LA; left atrium, LVEF; left ventricular ejection fraction, LDL; low-density lipoprotein, PP; pulse pressure, SBP; systolic blood pressure, T.chol; total cholesterol, TTE; transthoracic echocardiography, WC; waist circumference.

In subjects without MetS, age (*r* = 0.447, *p* < 0.001), systolic blood pressure (*r* = 0.194, *p* < 0.001). diastolic blood pressure (*r* = 0.078, *p* = 0.008), BMI (*r* = 0.188, *p* < 0.001), waist circumference (*r* = 0.181, *p* < 0.001), glucose (*r* = 0.165, *p* < 0.001), glycated hemoglobin (*r* = 0.146, *p* < 0.001), and LA size (*r* = 0.273, *p* < 0.001) showed correlation with E/e’ ratio.

### Multivariate analysis showing parameters associated with LVDD

The univariate analysis showed positive correlation between EAT and LVDD in subjects with MetS (OR 1.946, 95% confidence interval 1.308-2.896, *p* = 0.001), whereas in subjects without MetS, EAT did not show significant correlation with LVDD (*p* = 0.453).

To adjust other possible confounding variables, multivariate regression analysis was performed including age, systolic blood pressure, BMI, glucose and LDL cholesterol, as shown in Table 3. After adjusting for the covariables, EAT was still significantly associated with LVDD in subjects with MetS (OR 1.845, 95% CI 1.153-2.951, *p* = 0.011), whereas in subjects without MetS, such correlation was not found (*p* = 0.101).

**Table 3:** Multivariate analysis showing parameters associated with LV diastolic function

**Parameters**	**Exp (B)**	**95% confidence interval**	**p-value**
**Patients with metabolic syndrome**			
EAT by echocardiography, mm	1.845	1.153-2.951	0.011
Age, years	1.090	1.037-1.146	0.001
SBP, mmHg	1.034	1.000-1.069	0.051
BMI, kg/m2	1.002	0.832-1.206	0.986
Glucose, mg/dL	1.001	0.987-1.015	0.921
LDL cholesterol, mg/dL	0.999	0.985-1.013	0.847
**Patients without metabolic syndrome**			
EAT by echocardiography, mm	0.667	0.411-1.082	0.101
Age, years	1.105	1.057-1.154	<0.001
SBP, mmHg	1.037	1.007-1.068	0.015
BMI, kg/m2	1.175	1.006-1.371	0.041
Glucose, mg/dL	1.017	0.998-1.037	0.085
LDL cholesterol, mg/dL	0.993	0.979-1.007	0.302

BMI; body mass index, EAT; epicardial adipose tissue, LDL; low-density lipoprotein, SBP; systolic blood pressure

## Discussion

In this study we aimed to assess the association of EAT thickness with LVDD in subjects with MetS. Using two-dimensional echocardiography we demonstrated that EAT thickness is significantly associated with LVDD in subjects with MetS, and this association remains after adjusting for age, systolic blood pressure, BMI, blood glucose level, and LDL cholesterol. Importantly, we did not observe this association in subjects without MetS.

The echocardiographic measurement of EAT was proposed and validated by Iacobellis et al. [17,18]. Several studies have assessed epicardial fat using coronary CT or cardiac magnetic resonance imaging (MRI). However, measuring EAT by echocardiography has several advantages over CT or MRI. It is inexpensive, easier to access, and rapidly applicable especially within clinical practice, and has high reproducibility. Moreover, when compared to CT, echocardiography is radiation-free and free from contrast agent-related side effects. Considering these significant advantages and placed within the context of the current study population, which consisted of subjects without clinical signs or symptoms of heart disease who were self-referred for health exams, the data strongly suggests that the echocardiographic measurement of EAT is preferred to CT.

We found that EAT thickness was greater in subjects with MetS, consistent with previous studies [10,11,19]. Our data indicate that EAT thickness is associated with metabolic factors and is correlated with the main anthropometric and clinical parameters of metabolic syndrome [10,20,21]. This data is consistent with a previous study that reported EAT thickness increases with an increasing number of MetS components [11].

MetS is a cluster of several cardiometabolic risk factors and is eventually associated with cardiovascular disease. Visceral obesity and insulin resistance have been proposed as the main underlying mechanism. Both are strongly linked with hypertension, dyslipidemia and atherosclerotic coronary artery disease. In current study, we assessed functional aspect of myocardium rather than vascular disease. To avoid other possible confounding factors, we adjusted cardiometabolic risk factors using multivariate regression model. Our result still showed significant association between EAT and LV diastolic function in presence of MetS. Although the underlying mechanism is not fully understood yet, increased EAT, measured by either CT or echocardiography, has consistently shown independent correlation with impaired diastolic function. Cavalcante et al. reported that epicardial fat volume is an independent correlate of impaired diastolic function after accounting for associated comorbidities [9]. They studied 110 apparently healthy individuals, and found that epicardial fat volume adds increment predictive value for diastolic dysfunction.

Having similar properties and origin with visceral adipose tissue, EAT has been suggested as an active organ producing several proinflammatory and proatherogenic bioactive cytokines [22-24]. Also with its anatomic proximity to the heart without fascial protection inbetween, the possible local interaction via paracrine or vasocrine effect was suggested as a mechanism that causes coronary artery disease or LVDD [1,3,25,26].

As shown in our study, EAT thickness was independently associated with LVDD in subjects with MetS. LVDD is clinically significant and related to poor outcome. This was demonstrated in the longitudinal study of Olmsted County, in which increased all-cause mortality was significantly associated with presence of LVDD [27]. The significance was still present after adjusting for age, gender and ejection fraction.

### Limitations

There are several limitations in our study. First, the measurement of epicardial fat tissue by echocardiography can vary depending on the experience of the cardiologist. To minimize such inter- and intra-observer variability, we repeatedly compared images and had discussions among cardiologists in our center. From these group discussions an overall consensus was drawn on the interpretation of the images. Also, our cardiologists are all well-trained individuals with much experience in echocardiography. Second, since the study is performed as a cross-sectional study, we do not have clinical outcome data. Third, assessing additional biomarkers may help strengthen our findings and aid in the interpretation of our results.

## Conclusion

EAT thickness measured by echocardiography is independently associated with LV diastolic function in subjects with MetS. This correlation remained significant after adjusting for other known cardiometabolic risk factors, such as age, systolic blood pressure, BMI, blood glucose and LDL cholesterol.

## Competing interest

The authors declare that they have no competing interest.

## Authors’ contributions

HEP researched data contributed to discussion and wrote the manuscript, MKK acquired raw data, reviewed and edited the manuscript, SYC acquired raw data, contributed to the discussion and reviewed and edited the manuscript. All authors read and approved the final manuscript.

## Pre-publication history

The pre-publication history for this paper can be accessed here:

http://www.biomedcentral.com/1471-2261/14/3/prepub
